# Novel T-cell signature based on cell pair algorithm predicts survival and immunotherapy response for patients with bladder urothelial carcinoma

**DOI:** 10.3389/fimmu.2022.994594

**Published:** 2022-11-17

**Authors:** Xin Yan, Xiao Zhang, Hua-Hui Wu, Shao-Jie Wu, Xiao-Yu Tang, Tong-Zu Liu, Sheng Li

**Affiliations:** ^1^ Department of Urology, Zhongnan Hospital of Wuhan University, Wuhan, China; ^2^ Department of Biological Repositories, Cancer Precision Diagnosis and Treatment and Translational Medicine Hubei Engineering Research Center, Zhongnan Hospital of Wuhan University, Wuhan, China

**Keywords:** T cell, immunotherapy, bladder urothelial carcinoma, prognosis, drugs

## Abstract

**Background:**

T-cell–T-cell interactions play important roles in the regulation of T-cells’ cytotoxic function, further impacting the anti-tumor efficacy of immunotherapy. There is a lack of comprehensive studies of T-cell types in bladder urothelial carcinoma (BLCA) and T-cell-related signatures for predicting prognosis and monitoring immunotherapy efficacy.

**Methods:**

More than 3,400 BLCA patients were collected and used in the present study. The ssGSEA algorithm was applied to calculate the infiltration level of 19 T-cell types. A cell pair algorithm was applied to construct a T-cell-related prognostic index (TCRPI). Survival analysis was performed to measure the survival difference across TCRPI-risk groups. Spearman’s correlation analysis was used for relevance assessment. The Wilcox test was used to measure the expression level difference.

**Results:**

Nineteen T-cell types were collected; 171 T-cell pairs (TCPs) were established, of which 26 were picked out by the least absolute shrinkage and selection operator (LASSO) analysis. Based on these TCPs, the TCRPI was constructed and validated to play crucial roles in survival stratification and the dynamic monitoring of immunotherapy effects. We also explored several candidate drugs targeting TCRPI. A composite TCRPI and clinical prognostic index (CTCPI) was then constructed, which achieved a more accurate estimation of BLCA’s survival and was therefore a better choice for prognosis prediction in BLCA.

**Conclusions:**

All in all, we constructed and validated TCRPI based on cell pair algorithms in this study, which might put forward some new insights to increase the survival estimation and clinical response to immune therapy for individual BLCA patients and contribute to the personalized precision immunotherapy strategy of BLCA.

## Introduction

Bladder cancer (BC) is a malignant tumor that occurs on the bladder mucosa, which is the most common malignant tumor of the urinary system and one of the top ten common tumors among all cancer types (1). BC can occur at any age, even in children (1). Its incidence rate increases with age, and the high incidence age is 50–70 years old (1). According to the pathological types and histological classification of bladder cancer from the World Health Organization (WHO), bladder cancer can be categorized into several types, including bladder urothelial carcinoma (BLCA), bladder squamous cell carcinoma, bladder adenocarcinoma, and so on (2, 3). Among them, BLCA is the main type in BC, accounting for more than 90% of the total number of patients with BC (2). There are 573,278 new cases and 212,536 new deaths worldwide in 2020, according to GLOBOCAN 2020 (4). According to data contributed by the American Cancer Society, there will be about 81,180 newly confirmed BC patients and 17,100 more BC deaths in the United States in 2022 (5). The 5-year survival rate of BC patients depends on the severity of the disease. Generally speaking, a 5-year survival rate reaches 77%. When cancer spreads to the surrounding tissue or nearby lymph nodes or organs, the 5-year survival rate drops to 38%. What is more concerning, the 5-year survival rate is just 6% if BC distant metastasis occurs (2, 6). Overall, there is no significant improvement in the 5-year survival rate with improved early diagnosis and treatment of BLCA (6).

Immunotherapy is a type of cancer treatment that helps the immune system fight cancer (7). At present, more and more studies have indicated that tumors could be treated with immunotherapy effectively and safely (8–10). Although immunotherapy has been a hot research field and has actually brought hope to cancer patients, there are still some challenges in cancer immunotherapy (11). Clinical data show that although some cancer patients with overexpressed PD-L1 have a positive response to checkpoint inhibitors (CPI), a large part of them are not sensitive to CPI treatment (11). Even if some patients are reactive to CPI, the tumor will eventually recur (11, 12). So revealing the important molecular and cellular drivers of CPI treatment of primary and secondary immune escape and further improving the efficacy of immunotherapy must be a big challenge (11, 12). T cells are crucial effectors of anti-tumor immunity (13, 14). The anti-tumor efficacy of immunotherapy mainly depends on T cells’ cytotoxic function (15, 16). T-cell–T-cell interaction (including CD8+ T cell-Th17, CD8+ T cell-Treg, etc.) plays an important role in the regulation of T cells’ cytotoxic function (17, 18). The interaction between them can produce inflammation cytokines, alter the T-cell lineage landscape, and exhaust T-cells’ cytotoxic functions, ultimately leading to immune escape and resistance to immunotherapy (18–21). Therefore, exploring the T-cell–T-cell interaction function will be an essential strategy for better-conducting cancer immunotherapy.

In this research, through the utilization of meta-multiple gene expression cohorts, we first measured the T-cell infiltration level of each sample among these cohorts. Then, a T-cell pair (TCP) was established by using the 19 T-cell types collected from previous studies to simulate T-cell–T-cell interaction. We immediately developed and verified a T-cell-related prognostic index (TCRPI), which might play a crucial role in prognosis prediction, and immunotherapy efficacy monitoring.

## Materials and methods

### Collection of BLCA cohorts and the related clinical characterization

BLCA expression cohorts were retrieved from several public databases, including ArrayExpress (n = 2), Gene Expression Omnibus (GEO) database (n = 20), The Cancer Genome Atlas (TCGA) database (TCGA-BLCA), the cBioPortal website (MSKCC), and a large phase 2 trial (IMvigor210) (**Table S1**). Only a BLCA cohort with more than 20 BLCA samples and related survival and clinical information was collected and used in the present study. A total of 25 BLCA cohorts with survival information were included in our study. The related pieces of literatures on these cohorts are shown in **Table S1**. TCGA-BLCA microarray data were first downloaded from the TCGA database. The R package “DEseq.2” (22) was used for normalization and log2 transformation. For E-MTAB-1803, E-MTAB-4321, and MSKCC, the expression profiles were directly retrieved from the related website. The dataset IMvigor210 was also retrieved from its related website (http://research-pub.Gene.com/imvigor210corebiologies), and the normalization was also performed *via* “DEseq.2.” For the GEO cohorts platformed on Affymetrix, we generated the raw CEL files and applied the robust multichip average (RMA) method for normalization *via* the R package “affy” (23). For the GEO cohorts platformed on Illumina, we directly retrieved the normalized expression profiles from the GEO database. The package “sva” (24) in R software was used for the meta-entire cohort *via* the 25 collected cohorts, through the following three steps: data preprocessing; merging; and ComBat-adjusted handling. In total, 44 normal samples from 3,429 BLCA patients were included in the meta-entire cohort, 3,134 of had with complete clinical information.

### Application of cell pair algorithm to construct T-cell-related prognostic index

Based on a widely accepted literature review, a total of 19 T-cell types were collected in the present study (25–36). The gene sets of the same T-cell type from different literature were combined, and the non-T-cell types were excluded (**Table S2**). These T-cell types included CD4+ T cells, CD8+ T cells, cytotoxic T cells, etc. Because of the large number of publications about these T-cell types (**Table S2**), there was no doubt about the reliability and comprehensiveness of the 19 cell types. Then single-sample gene set enrichment analysis (ssGSEA) was applied to measure the T-cell infiltration level in each BLCA sample *via* the R package “GSVA” (37); in the present study, the normalized enrichment score (NES) was defined as the T-cell infiltration level. To avoid differences between different data cohorts, and improve the use of multiple cohorts, we then attempted to construct a T-cell-related prognostic index (TCRPI) *via* the cell pair algorithm; in the present study, a T-cell pair score (TCPs) was defined as 1 when the NES of T-cell type a was greater than the NES of T-cell type b. If the NES of T-cell type a was less than the NES of T-cell type b, the TCPs were assigned as 0. Some TCPs with constant values (zero or one) were removed for further analysis to minimize the biases caused by the platform-dependent preferential measurement. We immediately contained these TCPs for identifying prognostic TCPs by using survival analysis (the log-rank test method), based on the meta-entire cohort. Then we randomly divided the meta-entire cohort into a meta-training cohort, a meta-testing 1 cohort, a meta-testing 2 cohort, and a meta-testing 3 cohort according to a ratio of 1:1:1:1. *Via* the package “glmnet” (38) in R software, we conducted a least absolute shrinkage and selection operator (LASSO) penalized Cox regression analysis of these prognostic TCPs based on the meta-training cohort. The coefficients of the TCPs in the multivariate Cox proportional hazards model were used for TCRPI construction. The TCRPI of each BLCA patient was calculated by using the following formula:


$$\text{TCRPI}=\sum {}_{i=1}^{n}Coef_{i}\times TCPs_{i}$$

In which Coef represents the regression coefficient and TCPs represent the T-cell pair score of each TCP (T-cell pair). With the aim of splitting BLCA patients into high- and low-risk groups, we performed time-dependent receiver operating characteristic (ROC) curve analysis *via* the R package “survivalROC” (39), using the meta-training cohort. “5 years” was set as the time point for this analysis, and the TCPs showing the shortest distance between the ROC curve and the point were further determined as the grouping cutoff value in the present study. BLCA patients across the meta-training cohort, meta-testing 1 cohort, meta-testing 2 cohort, and meta-testing 3 cohort were separated into high-risk group and low-risk group (TCRPI-risk groups), respectively.

### Exploration of the association between TCRPI and survival, clinical characteristics, and genomic alterations of BLCA patients

To explore the prognostic value of the TCRPI, we then conducted a survival analysis by using the R package “survival.” The survival differences between TCRPI-risk groups were measured *via* the four cohorts. The prognostic role of the TCRPI was also validated *via* the meta-entire cohort and TCGA-BLCA data. A log-rank test was used to measure the survival difference; a P-value of <0.05 was considered statistically significant. Furthermore, we attempted to explore the clinical difference between low-and high-risk groups. Several clinical indicators such as age, gender, stage, and grade were included for assessing clinical differences. Fisher’s exact test was used to determine the statistical differences among the groups; a P-value of <0.05 was considered statistically significant. The single-nucleotide variant (SNV) data of the BLCA, which contained 407 samples, were also obtained from the TCGA database. The mutation landscape in BLCA patients grouped by TCRPI was presented by applying the R package “maftools” (40). Furthermore, we downloaded the Masked Copy Number Segment (MCNS) data by using the R package “TCGAbiolinks” (41). Genomic Identification of Significant Targets in Cancer (GISTIC) was applied to calculate the CNV variation type (gain or loss) and frequency of genes in the TCGA-BLCA cohort *via* the module GISTIC_2.0 on the GenePattern website (https://cloud.genepattern.org/). The R package “maftools” was used for the visualization of the CNV analysis results among the groups.

### Association between TCRPI and several mutation, and immune indices

The homologous recombination deficiency (HRD) score represents distinct types of genomic scarring and chromosomal instability caused by deoxyribonucleic acid repair deficiency and is thus regarded as a powerful biomarker of a given cancer (42). Therefore, the HRD scores for the TCRPI risk groups were calculated to compare their chromosomal instability with the Wilcox test. Besides, the mRNA stemness index (mRNAsi) has been identified as a novel predictor associated with stem-like indices and tumor prognosis (43). So that we collected the mRNAsi, mDNAsi, EREG-mDNAsi, and EREG-mRNAsi from previous research and further explored the differences among the TCRPI groups. Microsatellite instability (MSI) occurs because of functional defects in DNA mismatch repair in tumor tissue. MSI accompanied by DNA mismatch repair defects is an important tumor marker in the clinic (44). For the present study, we calculated the MSI for each sample from the TCGA-BLCA cohort *via* the R package “PreMSIm” (45). The index of cytolytic activity (CYT) is measured as a new biomarker of immunotherapy that could characterize the antitumor immunity of CD8+ cytotoxic T cells and macrophages. We then evaluated the CYT score for each sample across the TCGA-BLCA data; in detail, the CYT score was defined as the mean expression of PRF1 and GZMA (46). In addition, the difference between the TCRPI risk groups was also measured using the Wilcox test. A P-value of <0.05 was considered significant.

### Correlation of TCRPI with a set of bladder cancer signature and immunotherapy-predicted pathways

Several genetic signatures positively related to the clinical response to the anti-PD-L1 agent (atezolizumab) in BLCA were collected from Mariathasan’s study (47, 48). Some therapeutic signatures containing oncogenic pathways that might form non-inflammatory TME, gene characteristics related to targeted therapy, and gene signatures for radiotherapy response prediction were also retrieved. Then we explored the association between TCRPI and these bladder cancer signatures and immunotherapy-predicted pathways.

### Correlation of TCRPI with immune related features

To further explore the potential functions of TCRPI and provide an immune landscape for TCRPI, we calculated the immune score, stromal score, and tumor purity for each BLCA sample *via* the R package “ESTIMATE” (49). The T-cell dysfunction and exclusion (TIDE) used for the response to immunity treatment was also quantified for BLCA samples. The TIDE scores of BLCA samples from the TCGA-BLCA cohort were evaluated and retrieved *via* the website: http://tide.dfci.harvard.edu/. In addition, Thorsson et al. defined six immune-related subtypes (C1–C6) through a pan-cancer analysis of immune subtypes (50). C1 represented wound healing. C2 represented IFN-γ dominant. C3 represented inflammatory. C4 represented lymphocyte depleted. C5 represented immunologically quiet. C6 represented TGF-β dominant. We attempted to explore the differences between these subtypes and the TCRPI. The authors of this study also defined 56 molecular signatures associated with immune characteristics (50). Thus, we measured the correlation of TCRPI with these signatures. In view of the major significance of immune checkpoints (ICPs) and immunogenic cell death (ICD) modulators for tumor immunity, the associations of TCRPI with ICPs and ICD modulators were explored. Also, we collected several common bladder cancer biomarkers from previous studies, including CFH (51), FGFR3 (52), NMP22 (53), and TERT (54). We also compared the expression differences among TCRPI risk groups. The Wilcox method was used for statistical evaluation. ssGSEA was then applied to define the scores of 28 previously reported immune cells to measure the immune cell components among TCRPI risk groups.

### Role of TCRPI in response to anti-PD-1/L1 immunotherapy

We obtained two gene expression cohorts containing immunotherapy and survival information for this step. The expression matrix of cohort IMvigor210, patients who were treated with atezolizumab (an anti-PD-L1 antibody), was first normalized *via* the method we previously described. In total, we included 192 BLCA patients in the depth analysis. Furthermore, the expression matrix of cohort GSE78220, patients who were treated with pembrolizumab (an anti-PD-1 antibody), was also retrieved *via* the GEO database. We used the R package “limma” (55) for normalization. As a result, 27 patients, including their immunotherapy and survival information, were included. Our priority was to explore survival differences across the TCRPI-risk groups by implementing the “survival” package in R software. Moreover, the TCRPI difference between different response groups (CR, PR, PD, and SD) was explored, and the Kruskal–Wallis test was chosen in the present study. Moreover, using the R package “pROC” (56), we plotted receiver operating characteristic (ROC) curves to measure the prediction values of TCRPI for immunotherapy response. The area under the curve (AUC) was also evaluated to quantify the predictive value.

### Construction and verification of a composite TCRPI and clinical prognostic index (CTCPI)

To obtain the prediction value of TCRPI and compare the prognostic accuracy of TCRPI with other BLCA-related signatures, we collected three existing molecular signatures, including a 3-gene signature (57), a 6-gene signature (58), and a 12-gene signature (59). For these signatures, the concordance index (C-index) was calculated and considered the comparing standard. Moreover, TCRPI and several clinical indicators (gender, age, stage, and grade) were included for multivariable Cox proportional regression assessment by using the six cohorts. The features showing a significant value (P <0.05) were used to establish the CTCPI. Similarly, we also evaluated the C-index of the CTCPI *via* six cohorts. In addition, the prognostic performances of continuous TCRPI and CTCPI scores were compared using the C-index as the standard. The restricted mean survival (RMS) curve was used for visualizing the continuous C-index. RMS represents the life expectancy at 10 years for patients with different indicators (60). The higher the RMS time ratio, the greater the prognostic potential.

### Drug sensitivity exploring

Then we attempted to identify several novel therapeutic drugs that could provide novel choices for BLCA treatment. Based on related drug information from the Genomics of Drug Sensitivity in Cancer (GDSC) database (61), the R package “pRRophetic” (62) was used for drug response prediction. Ridge’s regression was first used to estimate the maximal inhibitory concentration (IC50) of each patient. Then 10-fold cross-validation was used to measure the accuracy of the estimation. We further divided the patients into high- and low-risk groups according to the level of TCRPI, and the Wilcoxon rank-sum test was used to measure significance. P <0.05 was considered significant.

### Statistical analysis

Continuous variables were analyzed using Student’s t-tests, U-tests, or nonparametric rank-sum tests. Categorical variables were analyzed using Chi-squared tests or Fisher’s exact tests. The T cell infiltration levels for BLCAs were estimated using ssGSEA *via* the R package “GSVA.” The cell pair algorithm was conducted as we mentioned above. Prognostic analyses were performed using Kaplan–Meier survival analysis and Cox univariate and multivariate analyses. Survival results were summarized using “forestplot” (R package). TCRPI was developed using the regression coefficient and T-cell pair score of TCPs. All data were analyzed with SPSS 22.0 for Windows (SPSS, Chicago, IL, USA) and R 4.0.3 (http://www.r-project.org/). The results with P <0.05 were considered statistically significant.

## Results

### TCRPI construction and its role in survival, clinical and invasiveness

**Figure 1:**
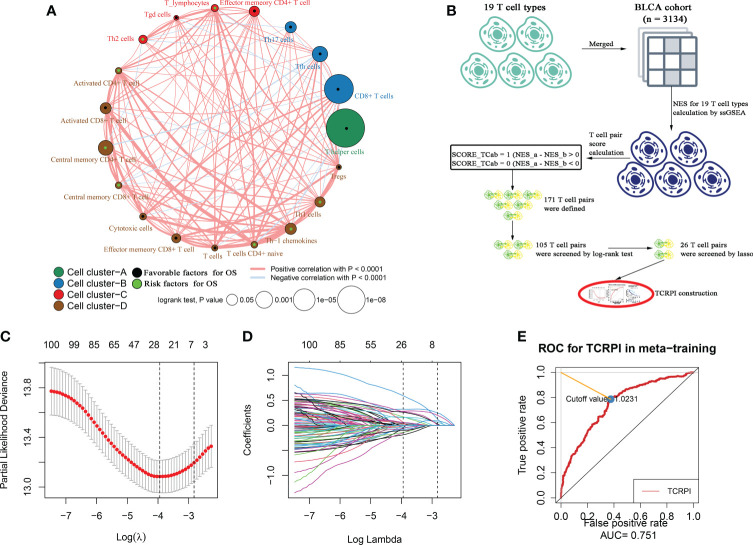
Survival landscape of the 19 T-cell types and T-cell related prognostic index (TCRPI) construction. **(A)** Cellular interaction and survival landscape of the 19 T-cell types. These T-cell types were separated into four clusters, the connection lines between cell types were divided into two kinds: red represents positive correlation, while blue represents negative correlation. The green dot in the T cell represents favorable factors for OS, while the black dot represents risk factors for OS. **(B)** The flow diagram of the TCRPI construction. **(C, D)** Plot of partial likelihood deviance for the 26 TCPs associated with survival in the training set. **(E)** Time-dependent ROC curve for TCRPI in the meta-training cohort at 5 years.

**Figure 2:**
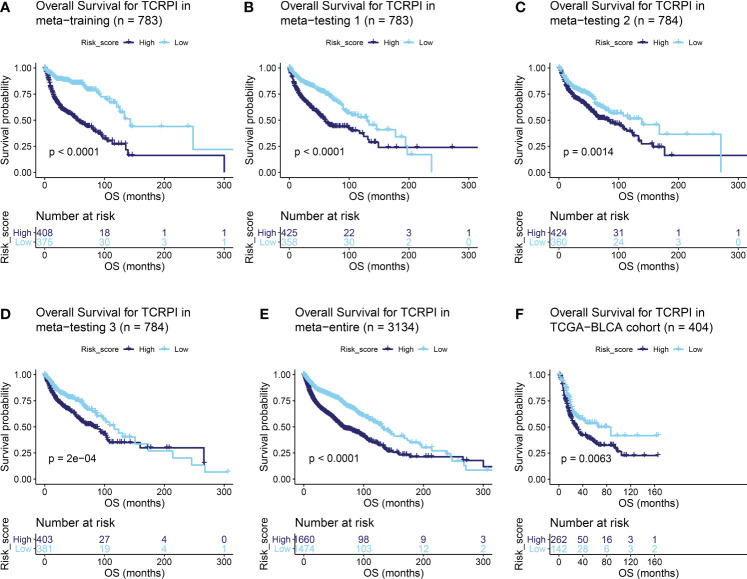
Survival difference across the TCRPI-high risk and TCRPI-low risk groups. **(A)** Overall survival curve for TCPRI in the meta-training cohort. **(B)** Overall survival curve for TCPRI in the meta-testing cohort 1. **(C)** Overall survival curve for TCPRI in the meta-testing cohort 2. **(D)** Overall survival curve for TCPRI in the meta-testing cohort 3. **(E)** Overall survival curve for TCPRI in the meta-entire cohort. **(F)** Overall survival curve for TCPRI in the TCGA-BLCA data.

**Figure 3:**
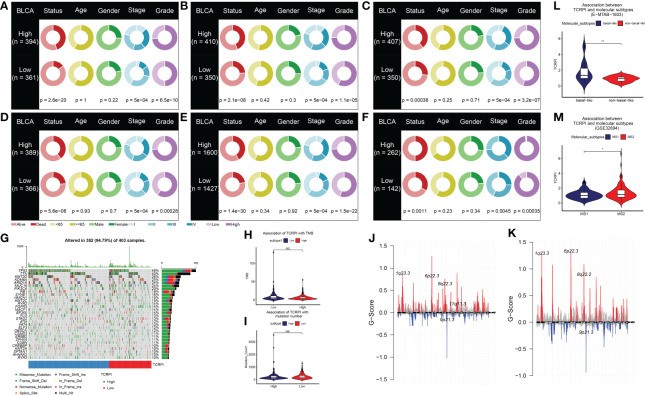
Association of TCRPI with clinical features and genomic correlations with TCRPI in the TCGA-BLCA data. The differences of clinical features (living status, age, gender, stage, and grade) across TCRPI-risk groups *via* meta-training cohort **(A)**, meta-testing cohort 1 **(B)**, meta-testing cohort 2 **(C)**, meta-testing cohort 3 **(D)**, meta-entire cohort **(E)**, and the TCGA-BLCA cohort **(F)**. **(G)** The oncoplot of the top 30 mutated genes that were associated with TCRPI. **(H)** Association of TCRPI with TMB. **(I)** Association of TCPRI with mutation number. **(J)** G-score distribution among the TCRPI-high risk group. **(K)** G-score distribution among the TCRPI-low risk group. **(L)** Association of TCPRI with molecular subtypes (basal-like, non-basal-like) using cohort E-MTAB-1803. **(M)** Association of TCPRI with molecular subtypes (MS1, MS2) using cohort GSE32894. NS, no significance, *P <0.05, **P <0.01.

A total of 19 T-cell types were collected and included in the present study. Then ssGSEA was used for the NES calculation of these T cells. Multivariate Cox analysis was obtained to measure the prognostic value of the 19 kinds of T cells (**Table S2**). **Figure 1A** described a comprehensive landscape of T-cell interactions, cell lineages, and their roles in the overall survival (OS) of BLCA patients. These T-cell types were separated into four clusters *via* the “hclust” method. Almost all of these T cells showed a strong positive correlation with each other. Several T-cell types showed negative correlations, such as central memory CD4+ T cells, which had a weak negative correlation with Tfh cells. As for the prognostic values of these T-cell types, these were inconsistent. We defined several kinds of T cells, including T helper cells (HR = 0.057, P <0.001), CD8+ T cells (HR = 0.044, P <0.001), and Tfh cells (HR = 0.189, P = 0.006) as favorable factors for the OS of BLCA patients. While some other T-cell types containing central memory CD4+ T cells (HR = 3.845, P = 0.008), Th-1 chemokines (HR = 1.366, P = 0.043) were identified as risk factors for BLCA’s OS. **Figure 1B** shows the TCRPI construction process. A total of 171 T-cell pairs (TCPs) were generated based on the NES of each T-cell type (**Table S3**). Then 105 TCPs identified by log-rank testing were further included in the LASSO algorithm (**Table S4**). A total of 26 TCPs were screened out (**Figures 1C, D**), 14 of which were generated by 21 T-cell types were picked out by multivariate Cox regression analysis and further used for TCRPI construction (**Figure S1A**, **Table S5**). According to the results of the time-dependent ROC curve at 5 years (**Figure 1E**, the area under the curve (AUC) = 0.751), BLCAs were categorized into two TCRPI0-risk groups (high and low) with the TCRPI cutoff value of 1.0231. *Via* the meta-training dataset (n = 783), it was concluded that BLCA patients in the TCRPI low-risk group (n = 375) had better OS compared to those in the TCRPI high-risk group (n = 408), as shown in **Figure 2A** (P <0.001). We reached the same conclusion *via* meta-testing cohort 1 (n of high-risk set = 425, n of low-risk set = 358, **Figure 2B**), meta-testing cohort 2 (n of high-risk set = 424, n of low-risk set = 360, **Figure 2C**), meta-testing cohort 3 (n of high-risk set = 403, n of low-risk set = 381, **Figure 2D**), meta-entire cohort (n of high-risk set = 1,660, n of low-risk set = 1,474, **Figure 2E**), and TCGA-BLCA cohort (n of high-risk set = 262, n of low-risk set = 142, **Figure 2F**). The TCRPI distribution of BLCA patients in the six cohorts is shown in **Figures S1B–M**. We further explored the clinical differences among TCRPI-risk groups (**Table S6**). In the meta-training dataset, patients classified into the TCRPI high-risk set had a higher stage (P <0.001) and grade (P <0.001) than these of patients classified into the low-risk group (**Figure 3A**). The same conclusion was reached by using meta-testing cohort 1 (**Figure 3B**), meta-testing cohort 2 (**Figure 3C**), meta-testing cohort 3 (**Figure 3D**), meta-entire cohort (**Figure 3E**), and the TCGA-BLCA cohort (**Figure 3F**). There was no difference in age or gender between high-risk group patients and low-risk group patients, which was further validated by using meta-testing cohort 1 (**Figure 3B**), meta-testing cohort 2 (**Figure 3C**), meta-testing cohort 3 (**Figure 3D**), meta-entire cohort (**Figure 3E**), and the TCGA-BLCA cohort (**Figure 3F**). Also, BLCA patients with lower TCRPI were less vulnerable to dying by comparison with those with higher TCRPI by using all six cohorts (**Figures 3A–F**). Higher tumor mutation burden (TMB), as well as higher somatic mutation rates, were correlated with stronger anti-cancer immunity. **Figure 3G** showed the mutation landscape of the top 30 high-frequency mutated genes in BLCA patients *via* TCGA data. There was a trend that the TCRPI-high risk group contained higher TMB compared to the TCRPI-low risk group (**Figure 3H**), as well as a higher number of mutated genes (**Figure 3I**). We also calculated the G-score for the two risk groups. Compared to the TCRPI-low risk group (**Figure 3K**), there was a higher mutation rate in the TCRPI-high risk group (**Figure 3J**). 6p22.3 was the idiosyncratic amplification type in the TCRPI-high risk group. It was known that the basal subtype was associated with more aggressive cancers; **Figure 3L** indicated that BLCA patients with the basal-like subtype had higher TCRPI levels compared with those with the non-basal-like subtype (P <0.01). Thus, the result indicated that the risk score level was positively associated with bladder cancer invasiveness. Lindgren et al. (63) defined two subtypes of BLCA (MS1, MS2), further proving that BLCAs of the MS2 subtype were correlated with aggressive growth and poor prognosis. We also suggested that BLCAs from the MS2 subtype had higher TCRPI levels compared with those from the MS1 subtype (**Figure 3M**, P <0.05). Taken together, TCRPI was positively associated with bladder cancer invasiveness, which might certainly predict aggressive cancer features.

### Evaluation of the TCRPI with other highly trustworthy indices

**Figure 4:**
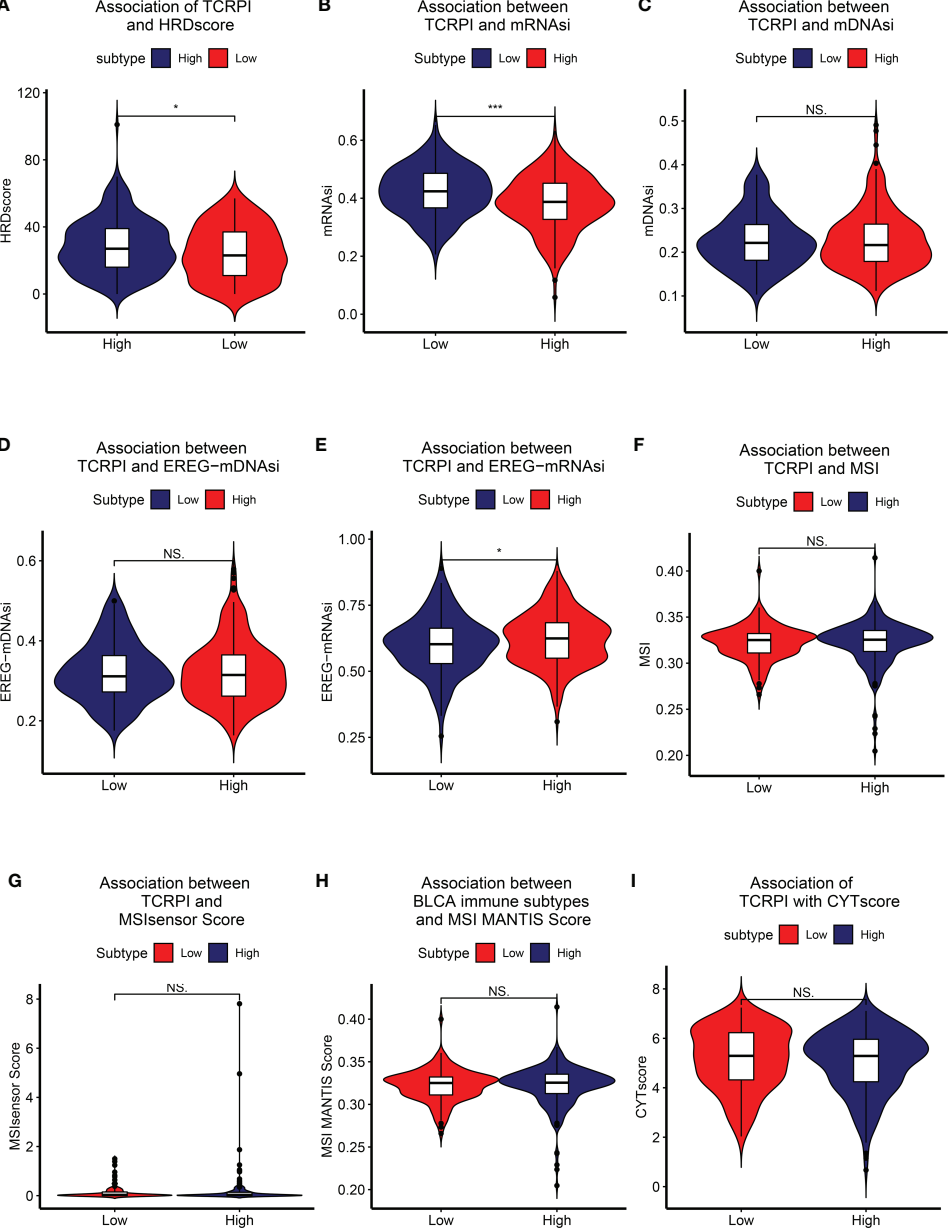
Association of TCRPI with other highly trustworthy indices. **(A)** HRDscore. **(B)** mRNAsi. **(C)** mDNAsi. **(D)** EREG-mDNAsi. **(E)** EREG-mRNAsi. **(F)** MSI. **(G)** MSI sensor score. **(H)** MSI MANTIS score. **(I)** CYT score. NS, no significance, *P <0.05, ***P <0.001.

HRD score represents distinct types of genomic scarring and chromosomal instability caused by deoxyribonucleic acid repair deficiency and is thus regarded as a powerful biomarker of a given cancer. Samples in the TCRPI-high risk group showed higher HRD scores compared to those in the TCRPI-low risk group (P <0.05, **Figure 4A**). As a novel predictor associated with stem-like indices and tumor prognosis, we found that the mRNAsi in the TCRPI-low risk group were higher than those in the TCRPI-high risk group (P <0.001, **Figure 4B**). As for mDNAsi (**Figure 4C**) and EREG-mDNAsi (**Figure 4D**), there were no statistical differences among TCRPI-risk groups. **Figure 4E** indicates that the EREG-mRNAsi in the TCRPI-low risk group were lower than those in the TCRPI-high risk group (P <0.05, **Figure 4E**). As a genomic characteristic for cancers, MSI is defined based on defective DNA mismatch repair. MSI has been identified as a meaningful marker for cancer diagnosis and treatment across a set of cancer types. A tendency has been proven that BLCAs in the TCRPI-high risk set showed greater MSI (**Figure 4F**), MSIsensor score (**Figure 4G**), and MSI MANTIS score (**Figure 4H**) than those in the TCRPI-low risk group. The cytolytic activity (CYT) score is a new index of cancer immunity calculated from the mRNA expression levels of GZMA and PRF1. We concluded that there was a trend that BLCA patients with higher TCRPI had higher CYT scores compared to those with lower TCRPI (**Figure 4I**). The details about these indices are shown in **Table S7**.

### Correlation of TCRPI with bladder cancer related pathways and immune-related features

**Figure 5:**
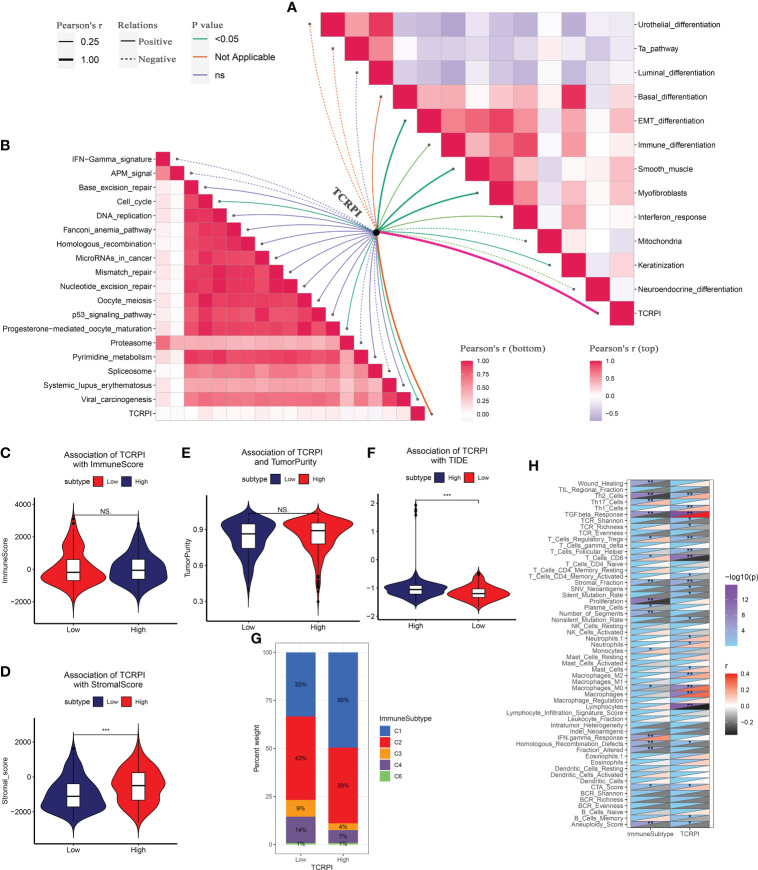
Correlation of TCRPI with bladder cancer-related pathways and immune-related features. **(A)** Association of TCRPI with bladder cancer related pathways. **(B)** Association of TCRPI with immunotherapy response related pathways. **(C)** Association of TCRPI with immune score. **(D)** Association of TCRPI with stromal score. **(E)** Association of TCRPI with tumor purity. **(F)** Association of TCRPI with TIDE. **(G)** Association of TCRPI with immune subtypes. **(H)** Association of TCRPI with 56 molecular signatures. NS, no significance, *P <0.05, **P <0.01, ***P <0.001.

**Figure 6:**
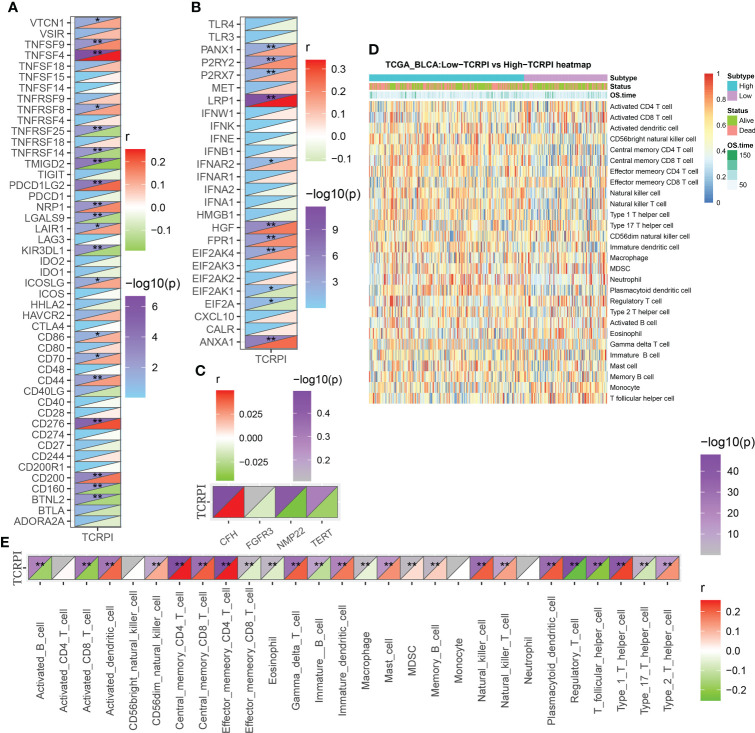
Immune landscape of TCRPI in BLCA. **(A)** Association of TCRPI with ICPs. **(B)** Association of TCRPI with ICD modulators. **(C)** Association of TCRPI with four bladder cancer biomarkers. **(D)** A heatmap showed the immune infiltration levels of the 28 immune cell types defined by ssGSEA. **(E)** Association of TCRPI with 28 immune cell types defined by ssGSEA. NS, no significance, *P <0.05, **P <0.01.

Then the correlation between TCRPI and previous bladder cancer-related pathways and immunotherapy-related pathways was explored. **Figure 5A** indicated that the TCRPI was positively related to EMT differentiation, immune differentiation, smooth muscle, myofibroblasts, interferon response, and keratinization. The TCRPI also showed a significantly negative association with mitochondria and neuroendocrine differentiation (**Figure 5A**). The TCRPI also showed associations with several pathways among the immunotherapy-related pathways (**Figure 5B**). The TCRPI was positively related to the cell cycle, progesterone-mediated oocyte maturation, and viral carcinogenesis (**Figure 5B**). There was no statistical difference in the immune score (**Figure 5C**) and tumor purity (**Figure 5E**) across TCRPI-risk groups. As shown in **Figure 5D**, patients classified into the TCRPI-high risk group showed a higher level of the stromal score (P <0.001) compared to the TCRPI-low risk group. A higher level of TIDE score indicated that patients were less likely to benefit from ICI treatment. **Figure 5F** concluded that BLCA patients in the TCRPI-low risk group were probably closer to benefiting from ICI treatment than they were from chemotherapy (**Figure 5F**, P <0.001). Patients in TCRPI-risk groups mainly overlapped with C1 and C2 (**Figure 5G**). Meanwhile, 14% of patients in the TCRPI-low risk group were classified into C4, and more patients in the TCRPI-high risk group were categorized into C1 compared to patients in the TCRPI-low risk group (**Figure 5G**). The relationship between the TCRPI and 56 molecular signatures was collected from previous studies. The TCRPI was positively associated with Th2 cells, Th1 cells, the TGFbeta response, neutrophils, mast cells, M2 macrophages, M0 macrophages, and the CTA score (**Figure 5H**). Furthermore, the TCRPI was negatively correlated with TCR Shannon, TCR richness, stromal faction, SNV neoantigens, silent mutation rate, and aneuploidy score (**Figure 5H**). In addition, we tried to explore the correlation between TCRPI and immune modulators, including ICPs and ICD modulators. **Figure 6A** showed the correlation of TCRPI with ICPs. The TCRPI was positively related to VTCN1, TNFSF9, TNFSF4, TNFRSF8, PDCD1LG2, NRP1, LAIR1, ICOSLG, CD86, CD70, CD44, CD276, and CD200, while negatively related to TNFRSF25, TNFRSF14, TMIGD2, LGALS9, KIR3DL1, CD160, and BTNL2 (**Figure 6A**). Moreover, the TCRPI was positively related to PANX1, P2RY2, P2RX7, LRP1, IFNAR2, HGF, FPR1, EIF2AK4, and ANXA1, while negatively correlated to EIF2AK1, and EIF2A (**Figure 6B**). Furthermore, the TCRPI showed no significant relationship with four bladder cancer biomarkers (**Figure 6C**). Then we explored the association between TCRPI and 28 immune cell types (**Figures 6D, E**). The TCRPI was positively related to 14 immune cell types (activated dendritic cell, central memory CD8 T cell, central memory CD4 T cell, etc.) meanwhile negatively related to nine immune cell types (Type 17 T helper cell, T follicular helper cell, etc.), whereas it was coincident with **Figure 5H**.

### The TCRPI could predict the immunotherapeutic benefit

**Figure 7:**
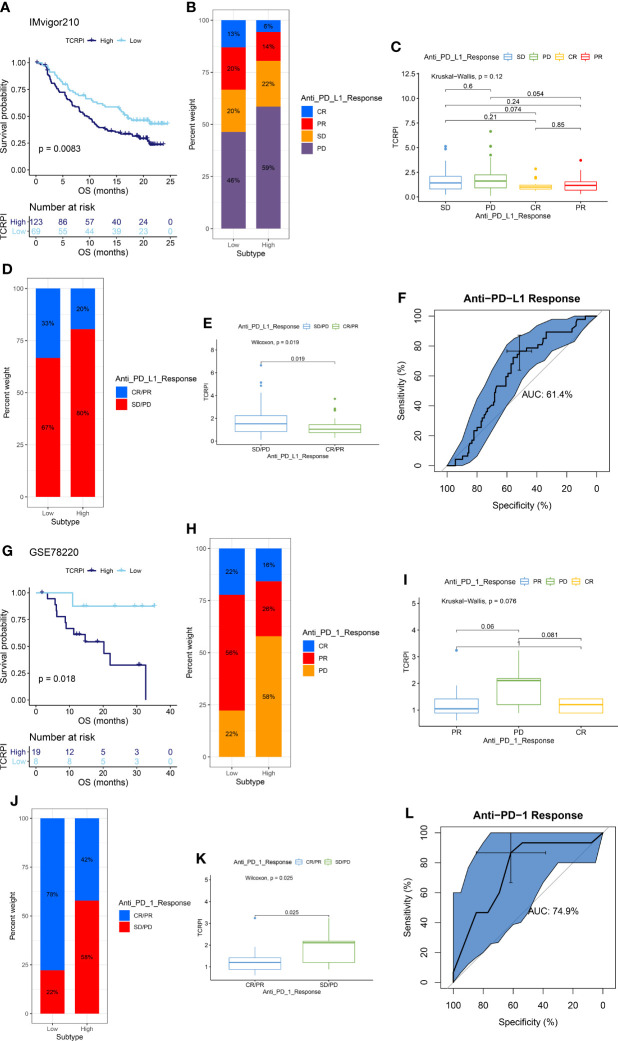
TCRPI is a prognostic biomarker and predicts immunotherapeutic benefit. **(A)** Kaplan–Meier curves for patients with high (n = 125) and low (n = 67) TCRPI in the IMvigor210 cohort. **(B)** Rate of clinical response [complete response (CR)/partial response (PR) and stable disease (SD)/progressive disease (PD)] to anti-PD-L1 immunotherapy in high or low TCRPI groups in the IMvigor210 cohort. **(C)** Distribution of TCRPI in groups with different anti-PD-L1 clinical response statuses. **(D)** Rate of clinical response [complete response (CR), partial response (PR), stable disease (SD) and progressive disease (PD)] to anti-PD-L1 immunotherapy in high or low TCRPI groups in the IMvigor210 cohort. **(E)** Distribution of TCRPI in groups with different anti-PD-L1 clinical response statuses. **(F)** ROC curve measuring the predictive value of the TCRPI. **(G)** Kaplan–Meier curves for patients with high (n = 10) and low (n = 17) TCRPI in the GSE78220 cohort. **(H)** Rate of clinical response [complete response (CR)/partial response (PR) and stable disease (SD)/progressive disease (PD)] to anti-PD-1 immunotherapy in high or low TCRPI groups in the GSE78220 cohort. **(I)** Distribution of TCRPI in groups with different anti-PD-1 clinical response statuses. **(J)** Rate of clinical response (complete response (CR), partial response (PR), stable disease (SD) and progressive disease (PD)) to anti-PD-1 immunotherapy in high or low TCRPI groups in the GSE78220 cohort. **(K)** Distribution of TCRPI in groups with different anti-PD-1 clinical response statuses. **(L)** ROC curve measuring the predictive value of the TCRPI.

Immunotherapies represented by PD-L1 and PD-1 blockade have undoubtedly emerged as major breakthroughs in cancer treatment. In IMvigor210, BLCA patients in the TCRPI-high set (n = 123) had meaningfully shorter survival (P = 0.0083, **Figure 7A**), compared to BLCAs classified into the TCRPI-low set (n = 69). The predictive value of the TCRPI for anti-PD-L1 immune therapy was further confirmed (**Figures 7B–E**). Patients divided into the TCRPI-low risk group were more likely to be helped by anti-PD-L1 therapy (**Figures 7B, D**), which was validated by the Kruskal–Wallis test (P = 0.12, **Figure 7C**) and the Wilcox test (P = 0.019, **Figure 7E**). Patients with CR states showed the lowest TCRPI level among all the anti-PD-L1 response states (**Figure 7C**). TCRPI was indicated to be a predictive biomarker for immunotherapy benefits (AUC: 0.614, **Figure 7F**) through the efficacy of anti-PD-L1 therapy exploration. Furthermore, by cohort GSE78220, we also explored whether TCRPI could play a role in the response to anti-PD-1 treatment. Patients divided into the TCRPI-high set showed worse survival by comparison with the TCRPI-low set (P = 0.018, **Figure 7G**). Similarly, patients in the TCRPI-low risk group could respond to anti-PD-1 immunotherapy better compared with those classified into the TCRPI-high set (**Figures 7H, J**), as concluded by the Kruskal–Wallis test (P = 0.076, **Figure 7I**) and the Wilcox test (P = 0.025, **Figure 7K**). The TCRPI was then concluded to be a suitable prediction application for anti-PD-1 therapy benefits (AUC: 0.749, **Figure 7L**). When taken together, the present study obviously demonstrated that TCRPI was correlated with anti-PD-L1/PD-1 immune therapy response, which might make a contribution to the prediction of response to immunotherapy.

### Predictive value comparison of TCRPI with several molecular signatures

**Figure 8:**
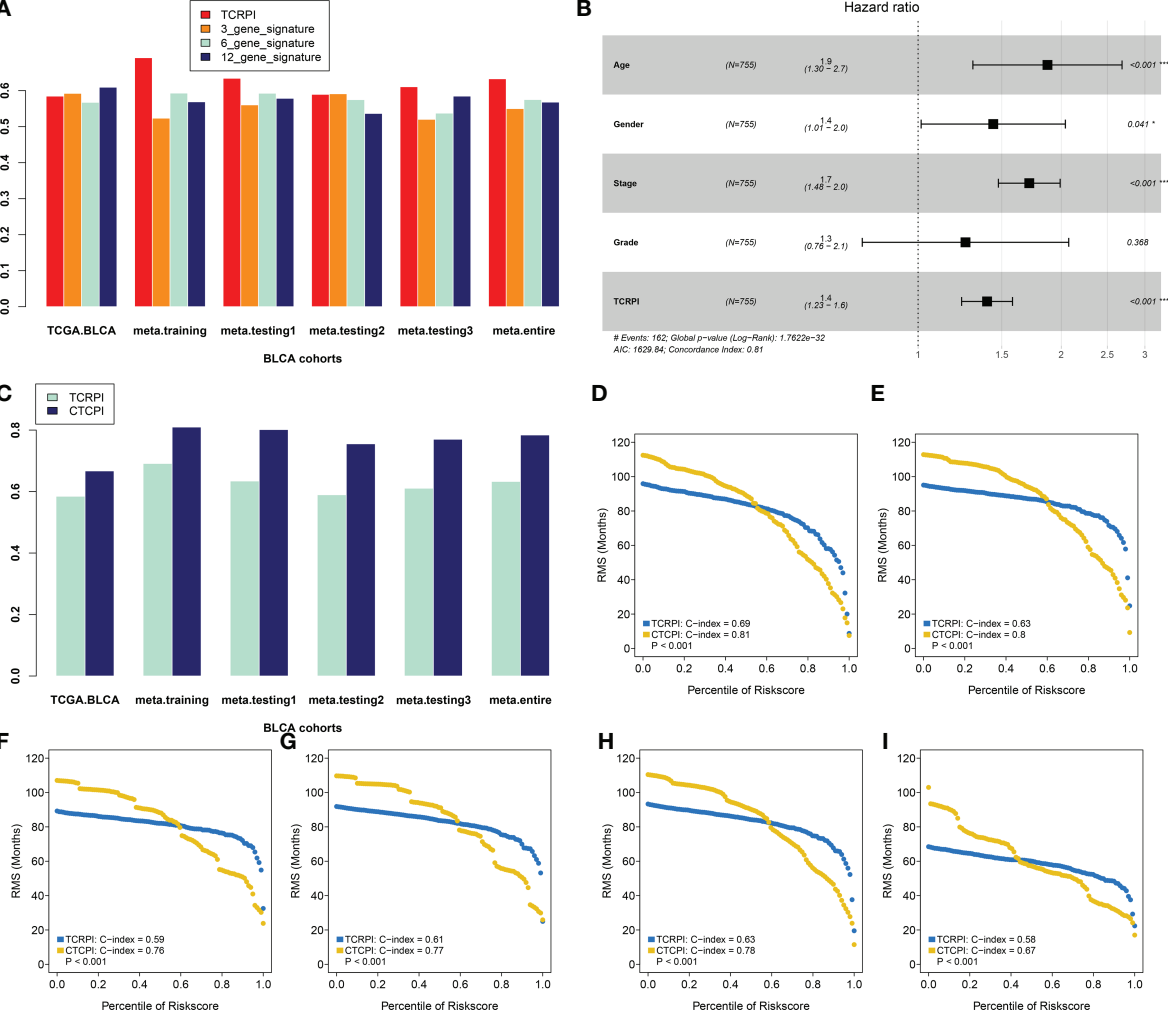
Construction of a Composite TCRPI and clinical prognostic index (CTCPI). **(A)** C-index comparison between TCRPI, 3-gene signature, 6-gene signature, and 12-gene signature. **(B)** Forest plot for the Hazard Ratios (HRs) of high vs low TCRPI risk groups *via* a meta-training cohort. **(C)** C-index comparison between TCRPI and CTCPI. Restricted mean survival (RMS) curves for continuous TCRPI and CTCPI in the meta-training cohort **(D)**, meta-testing cohort 1 **(E)**, meta-testing cohort 2 **(F)**, meta-testing cohort 3 **(G)**, meta-entire cohort **(H)**, and the TCGA-BLCA cohort **(I)**. NS, no significance, *P <0.05, ***P <0.001.

To determine whether the TCRPI was better than the previous prognostic signatures, three multiple gene signatures were collected and included in the present study. As shown in **Figure 8A**, the TCRPI showed the best prognosis prediction potential compared to the 3-gene signature, 6-gene signature, and 12-gene signature in the meta-training cohort, meta-testing cohort 1, meta-testing cohort 3, and meta-entire cohort. But in the TCGA-BLCA cohort, the TCRPI did not perform as well as the 12-gene signature.

### Construction of CTCPI and its prognostic role

To maximize the use of the TCRPI in the prognosis prediction of BLCA patients, we immediately contained the TCRPI and several essential clinical factors (gender, age, stage, grade) in the multivariable Cox analysis *via* a meta-training cohort (**Figure 8B**). Age (HR = 1.9, P <0.001) and Stage (HR = 1.7, P <0.001) were then screened out and generated with TCRPI (HR = 1.4, P <0.001) to construct CTCPI. The prognostic value of age and stage were also validated by meta-testing cohort 1 (**Figure S2A**), meta-testing cohort 2 (**Figure S2B**), meta-testing cohort 3 (**Figure S2C**), meta-entire cohort (**Figure S2D**), and the TCGA cohort (**Figure S2E**). Based on these results of the Cox test, the CTCPI of BLCA patients was defined as Age ∗ 0.627 + Stage ∗ 0.538 + TCRPI ∗ 0.334. Significantly improved estimation of survival was realized by CTCPI (**Figure 8C**), validated by RMS curves in the meta-training cohort (mean C-index: CTCPI: 0.81, TCRPI: 0.69, P <0.001, **Figure 8D**), meta-testing cohort 1 (mean C-index: CTCPI: 0.80, TCRPI: 0.63, P <0.001, **Figure 8E**), meta-testing cohort 2 (mean C-index: CTCPI: 0.76, TCRPI: 0.59, P <0.001, **Figure 8F**), meta-testing cohort 3 (mean C-index: CTCPI: 0.77, TCRPI: 0.61, P <0.001, **Figure 8G**), meta-entire cohort (mean C-index: CTCPI: 0.78, TCRPI: 0.63, P <0.001, **Figure 8H**), and TCGA-BLCA cohort (mean C-index: CTCPI: 0.67, TCRPI: 0.58, P <0.001, **Figure 8I**).

### Response to drug response

**Figure 9:**
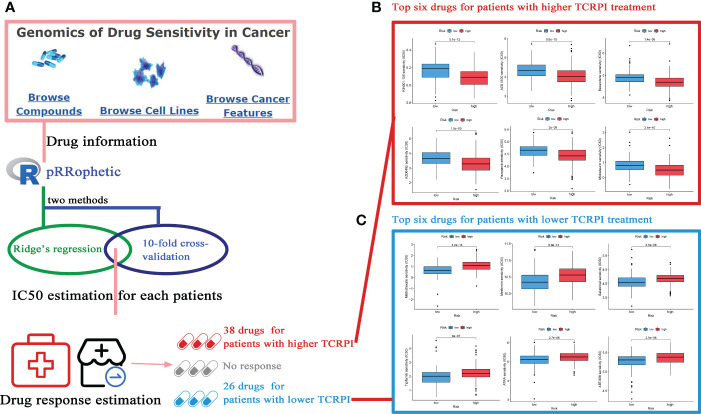
Potential drugs for BLCA treatment exploration. **(A)** The flow diagram of the IC50 estimation and drug sensitivity estimation (38 drugs for patients with a higher TCRPI, 26 drugs for patients with a lower TCRPI. **(B)** The top six drugs for BLCA patients with higher TCRPI treatment. **(C)** The top six drugs for BLCA patients with lower TCRPI treatment.

After categorizing BLCAs into TCRPI-high- and TCRPI-low-groups, the relationship between TCRPI and medical treatment was examined. As mentioned above (**Figure 9A**), drugs from the GDSC database with reported therapeutic potential for cancer were analyzed. As the results indicated, BLCAs in the TCRPI-high group were more sensitive to 38 drugs, while BLCAs in the TCRPI-low group were more sensitive to 26 drugs (**Table S9**). Moreover, the top six drugs for BLCAs in the TCRPI-high group, including KIN001.135, AZD.0530, Bexarotene, AZD6482, Pazopanib, and Midostaurin, are shown in **Figure 9B**. We also showed the top six drugs for BLCAs in the TCRPI-low group in **Figure 9C**. The results indicated that TCRPI could predict increased sensitivity to these therapeutic drugs in BLCA patients.

## Discussion

T cells are the most numerous type with complex functions in lymphocytes (64). For instance, helper T cells have the function of assisting humoral immunity and cellular immunity, suppressor T cells could inhibit cellular immunity and humoral immunity, and cytotoxic T cells have the function of killing target cells (64). They are brave soldiers formed in the body to resist disease, infection, and tumors (65). T cells have been proven to be essential effectors in anti-tumor immunity. T-cell–T-cell interactions such as CD8+ T cell-Th17 and CD8+ T cell-Treg could regulate the cytotoxic function of T cells, further impacting the anti-tumor efficacy of immunotherapy. Some recent studies of BLCA have also focused on T cells. Oh et al. concluded CD4 T cells could kill autologous cancers in an MHC class II-dependent fashion, which could be suppressed by regulatory T cells (66). A cytotoxic CD4 T cell-related index was further constructed by them, predicting clinical response for 244 metastatic bladder cancer patients treated with anti-PD-L1 (66). Liu et al. indicated that intratumoral TIGIT + CD8 + T-cell infiltration determined a worse prognosis and immune evasion for BLCA patients (67). Eckstein et al. established a cytotoxic T-cell-related index that could predict survival benefits in BLCAs after radical cystectomy and adjuvant chemotherapy (68). The above research proved the importance of T cells in survival, anti-tumor immunity, and therapy response in BLCA. But none of them considered the integrity and comprehensive interaction of T cell types. Since there were a set of T-cell types, it was of urgent need to give a landscape of these T-cell types in BLCA. A total of 19 T-cell types were collected from a previous study, and the infiltration level of each of them was evaluated for over 3,100 samples from public databases by using ssGSEA. The ssGSEA algorithm scored the individual samples independently without considering other samples in gene expression cohorts, which could overcome the calculation error caused by the multiple platforms of cohorts. Then a T-cell pair algorithm was applied to measure the interactions of T-cell types and further construct a T-cell-related prognostic index (TCRPI). Also, the cell pair algorithm only involves pairwise comparison within the cell infiltration level cohort of a sample, which allowed us to use samples from multiple platforms. The impact of TCRPI on survival was then measured. We found that BLCA patients with higher TCRPI were less likely to show better survival. This indicated that TCRPI was a risk indicator for survival and prognosis in BLCA patients. The clinical difference among TCRPI-risk groups was also explored; BLCA patients categorized into the TCRPI low-risk group were less likely to die and progress to an advanced stage or high-grade BLCA. The 5-year survival rate for advanced BLCA was as low as 4%, which was consistent with the present study.

HRD leads to a defect in the repair pathway of double-strand breaks, causing a high sensitivity to PARP inhibitors (PARPi), which have been used as a biomarker for therapy decision-making (69). As a recent study reported, the HRD score could also predict response to neoadjuvant chemotherapy in some cancer types, such as triple-negative breast cancer (70). BLCA patients in the TCRPI-high risk group showed higher HRD scores. mRNAsi was a novel predictor associated with stem-like indices and tumor prognosis. In the present study, we found that TCRPI was negatively associated with mRNAsi, which represented mRNAsi as a protective factor for the survival of patients with BLCA. Pan et al. concluded higher mRNAsi in BLCA were associated with better overall survival (71), which was consistent with our result. MSI occurs because of functional defects in DNA mismatch repair in tumor tissue. MSI accompanied by DNA mismatch repair defects is an important tumor marker in the clinic. The CYT index is measured as a new biomarker of immunotherapy that could characterize the antitumor immunity of CD8+ cytotoxic T cells and macrophages. In the present study, we also tried to explore the relationship between TCRPI and them. No significant results were obtained. Perhaps because the TCRPI might not act on DNA mismatch repair. We have conducted studies showing that the TCRPI could predict immunotherapeutic benefits; thus, we thought the TCRPI impact on immunotherapy could be achieved without targeting PRF1 and GZMA.

Then we attempted to characterize the immune landscape across TCRPI-risk groups. The TCRPI might regulate some bladder cancer-related pathways and immune related features; in detail, the TCRPI was positively related to EMT differentiation, immune differentiation, smooth muscle, myofibroblasts, interferon response, keratinization, cell cycle, progesterone-mediated oocyte maturation, and viral carcinogenesis. The TCRPI also showed a significantly negative association with mitochondria and neuroendocrine differentiation. A higher level of TIDE score indicated that patients were less likely to benefit from ICI treatment. **Figure 5F** concluded that BLCAs in the TCRPI-low risk set might more likely benefit from ICI treatment. The correlation of the intrinsic immune escape mechanism with the TCPRI in the BLCA was also explored. The TCRPI was concluded to be associated with some ICPs, including VTCN1, TNFSF9, TNFSF4, TNFRSF8, PDCD1LG2, NRP1, LAIR1, ICOSLG, CD86, CD70, CD44, CD276, and CD200, which indicated that the TCRPI could be an effective indicator for immune checkpoint blockage (ICB) therapy.

Tumor immunotherapy is a novel therapeutic option for controlling and eliminating tumors by restarting and maintaining the tumor immune cycle and restoring the normal anti-tumor immune response (72). Because of its excellent curative effect and innovation, it was rated as the most important scientific breakthrough of the year by the magazine Science in 2013. These days, an increasing number of investigators have concentrated on exploring new immune-related prognostic indicators and therapeutic targets for immunotherapy. Further integrated analyses indicated that the TCRPI was related to response to anti-PD-L1/PD-1 immune therapy, which might help improve the predictive strategy for immunotherapy. To maximize the application of the TCRPI in the prognosis prediction of BLCA patients, we then constructed a CTCPI considering both the TCRPI and several clinical features. The C-index improved from 0.69 to 0.81, which significantly improved the estimation of BLCA’s survival.

There were some limitations to our study. Firstly, although the TCPRI could distinguish well between high- and low-risk groups, it was not clear if it could show positive performance as we expected in clinical trials. Thus, we will further apply and test this signature in clinical judgment for the prognosis of BLCAs. Secondly, the roles of the TCRPI and T cells in BLCA progression and prognosis must be explored and validated by using *in vivo* and *in vitro* experiments. Thirdly, because the AUC values for the TCRPI for immunotherapy were lower than 0.75, we will perfect the TCRPI and validate its immunotherapy predictive value by using our own data in the near future.

All in all, the work in this study put forward some new insights to increase the survival estimation and clinical response to immune therapy for individual BLCA patients through the comprehensive analysis of T-cell types, which might contribute to the personalized precision immunotherapy strategy of BLCA over the next few decades.

## Conclusions

All told, we constructed and verified a T-cell-related prognostic index (TCRPI) in this study, which might be a useful tool for prognosis prediction of BLCA and contribute to identifying patients suitable for immunotherapy. A comprehensive evaluation of the interactions of T cells in BLCA would help us improve our cognition of the infiltration characteristics and functions of T cells. This would guide more effective immunotherapy strategies.

## Data availability statement

All database generated for this study are included/have their accession numbers included in the article.

## Author contributions

SL and T-ZL designed this research. SL, T-ZL, and XY organized the processing flow. XY completed the whole analytic process of this study. XY, XZ, and H-HW organized and presented the results. XY contributed to the writing of the manuscript. All authors listed have made a substantial, direct, and intellectual contribution to the work and approved it for publication.

## Funding

This work was supported by the Zhongnan Hospital of Wuhan University Science, Technology and Innovation Seed Fund (znpy2019077) and Translational Medicine and Interdisciplinary ResearchJoint Fund of Zhongnan Hospital of Wuhan University (Grant No.ZNJC202232).

## Acknowledgments

We are grateful for the TCGA and GEO databases developed by the National Institutes of Health (NIH), the cBioPortal website developed by Memorial Sloan Kettering Cancer Center (MSK), and the ArrayExpress database developed by EMBL’s European Bioinformatics Institute. We also want to acknowledge the funding that supports us.

## Conflict of interest

The authors declare that the research was conducted in the absence of any commercial or financial relationships that could be construed as a potential conflict of interest.

## Publisher’s note

All claims expressed in this article are solely those of the authors and do not necessarily represent those of their affiliated organizations, or those of the publisher, the editors and the reviewers. Any product that may be evaluated in this article, or claim that may be made by its manufacturer, is not guaranteed or endorsed by the publisher.
