# Supplementary figures and images for: Novel T-cell signature based on cell pair algorithm predicts survival and immunotherapy response for patients with bladder urothelial carcinoma
Source: Front Immunol. 2022 Nov 17;13:994594. doi: 10.3389/fimmu.2022.994594 (PMC9712189; doi:10.3389/fimmu.2022.994594)

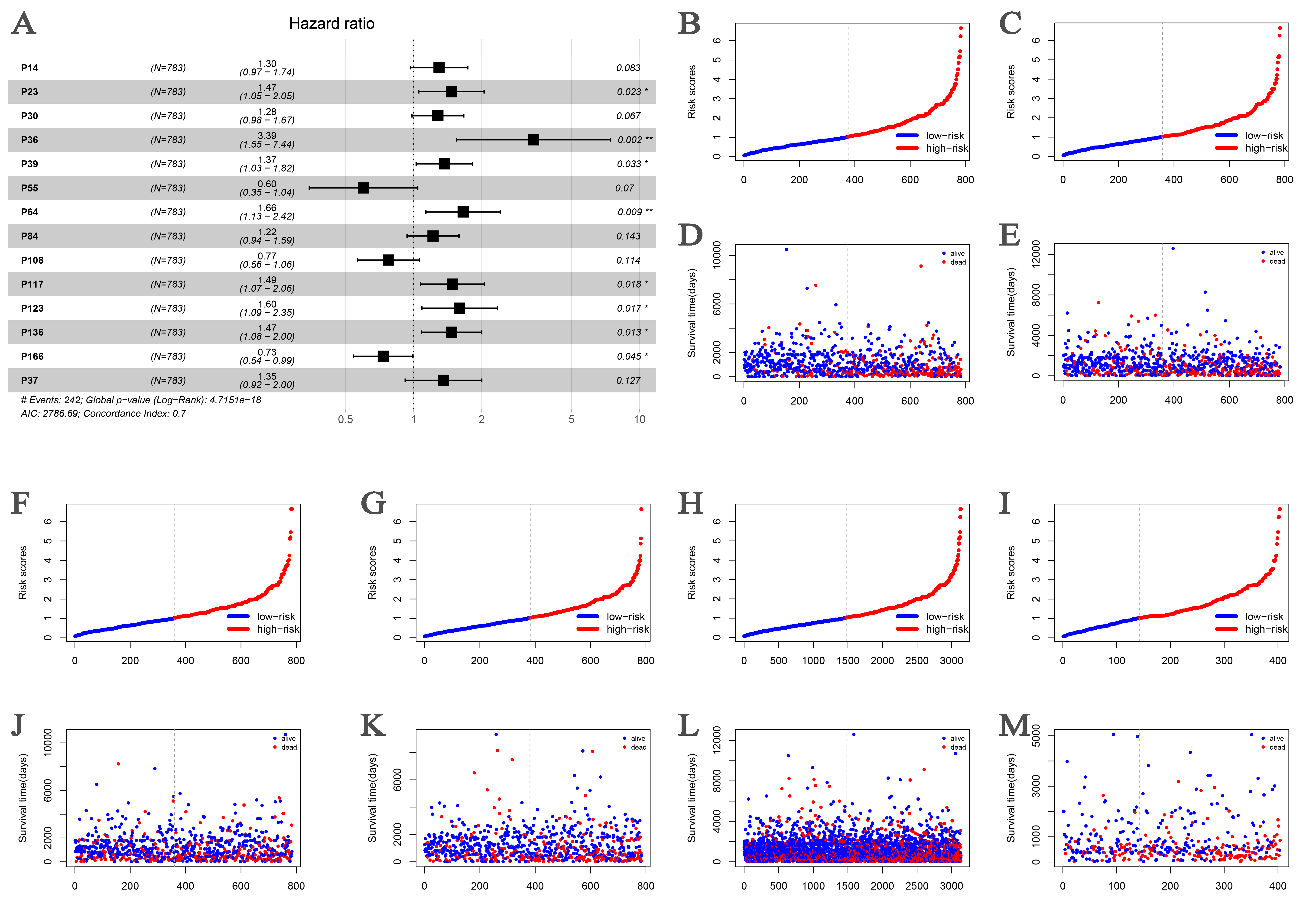

Supplement: Supplementary file 1 [file Image_1.tif]

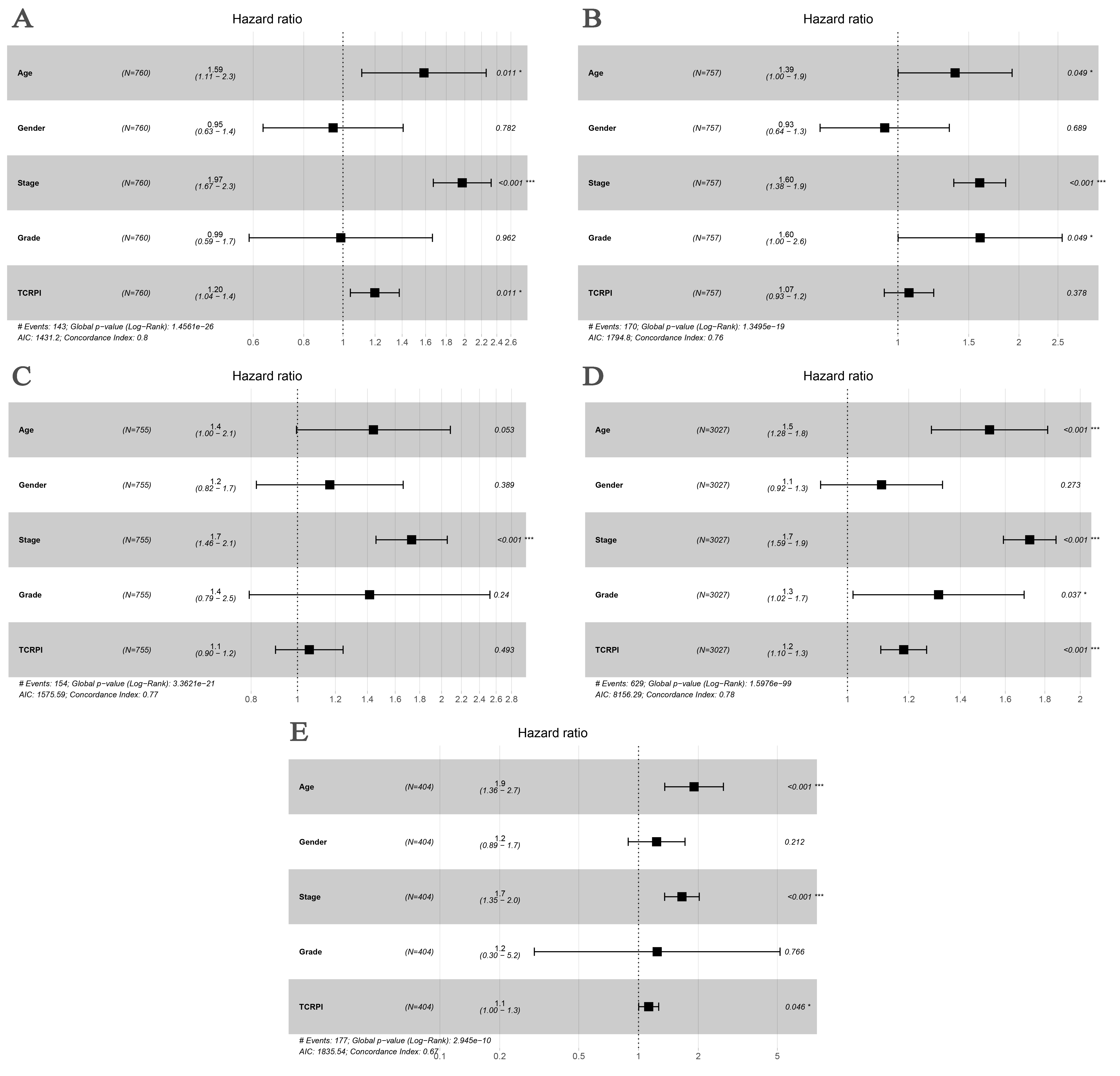

Supplement: Supplementary file 2 [file Image_2.tif]
